# Effect of Da-Cheng-Qi Decoction on Pancreatitis-Associated Intestinal Dysmotility in Patients and in Rat Models

**DOI:** 10.1155/2015/895717

**Published:** 2015-03-02

**Authors:** Jianlei Zhao, Cejun Zhong, Zhiyu He, Guangyuan Chen, Wenfu Tang

**Affiliations:** ^1^Department of Pharmacology, School of Preclinical and Forensic Medicine, West China Medical Center, Sichuan University, Chengdu 610041, China; ^2^Center of Infectious Diseases, West China Hospital, Sichuan University, Chengdu 610041, China; ^3^Department of Integrated Traditional Chinese and Western Medicine, West China Hospital, Sichuan University, Chengdu 610041, China

## Abstract

The impairment of intestinal motility and related infectious complications are the predominant clinical phenomenon in patients with severe acute pancreatitis (SAP). We aimed to investigate the effects of Da-Cheng-Qi decoction (DCQD) on the gastrointestinal injury in SAP patients and the potential mechanism involved in rats. DCQD was enema administered to 70 patients for 7 days in West China Hospital. Mortality and organ failure during admission were observed and blood samples for laboratory analysis were collected. We also experimentally examined plasma inflammatory cytokines in rat serum and carried the morphometric studies of the gut. Intestinal propulsion index and serum and tissue vasoactive intestinal peptide (VIP) were also detected. Though DCQD did not affect the overall incidence of organ failure, it shortened the average time of paralytic intestinal obstruction and decreased the morbidity of infectious complications in patients with SAP. Compared with untreated rats, the DCQD lowered the levels of proinflammatory cytokine and decreased the mean pathological intestinal lesion scores. The VIP level in intestinal tissue or serum in DCQD group was obviously lowered and intestinal propulsion index was significantly improved. In conclusion, DCQD has good effect on pancreatitis-associated intestinal dysmotility in patients and in rat models.

## 1. Introduction

Severe acute pancreatitis (SAP) is a serious systemic disease with high mortality of about 15–30% with high morbidity of acute lung injury, acute kidney injury, and impairment of intestinal motility [1]. A large scale retrospective placebo-controlled clinical trial indicated that Da-Cheng-Qi decoction (DCQD) could exert significant effects on reducing mortality in patients with SAP [2]. Our previous study also demonstrated the beneficial effect of DCQD against lung injury in SAP through promoting the gastrointestinal motility [3]. The impairment of intestinal motility is the predominant clinical change at the early phase of the disease that might be relative to the systematic inflammatory response and the infectious complications at the late stage in patients with SAP [4, 5]. It is accepted that mortality in the late phase of SAP is primarily due to infectious complications, particularly infected pancreatic necrosis. Inflammatory mediators burst in the course of acute pancreatitis or the changed gastrointestinal hormones could alter the gastrointestinal motility in a direct or indirect way [6, 7]. A number of gastrointestinal hormones such as vasoactive intestinal peptide (VIP), by acting on the specific VIP_2_ receptors on the surface of gastrointestinal smooth muscle, played an important role in the regulation of gastrointestinal motility [8]. On the other hand, the inflammation of pancreas is characterized by the release of proinflammatory cytokines such as tumor necrosis factor-*α* (TNF-*α*), interleukin-1*β* (IL-1*β*), and interleukin-6 (IL-6) [9]. It is important to improve the gastrointestinal motility through inhibiting the inflammatory response. Unfortunately, there is no effective treatment for the inflammatory response. Prophylactic antibiotics, once routinely used in the treatment of SAP, showed no statistical differences in reducing the risk of infectious complications [10–13]. So, appropriate phytotherapy aimed at controlling the pathophysiological mechanisms that lead to enteroparalysis or inflammation may develop an effective treatment for SAP. Our previous studies showed Cheng-Qi decoction (DCQD) had some anti-inflammation effect in patients with acute pancreatitis. However, the potential effect of DCQD on the intestinal dysmotility in the course of acute pancreatitis is to be elucidated. DCQD is composed of Dahuang (*Radix et Rhizoma Rhei*), Houpu (*Magnolia officinalis Rehd*), Zhishi (*Fructus Aurantii Immaturus*), and Mangxiao (*Natrii Sulfas*) with ratio of 1 : 1 : 1 : 1 in weight. They are traditionally used as purgatives for the treatment of constipation and for clearing internal heat in the stomach and intestine [14]. Its efficacy is similar to the modern theory of “selective decontamination of the digestive tract in the management of SAP.” But it remains unclear whether DCQD could improve SAP-associated alterations of gastrointestinal motility and the related inflammatory response. So, the study aimed to explore the effect of DCQD on the gastrointestinal motility in SAP. Moreover, to clarify the mechanism involved, VIP, inflammatory mediators, and the rate of intestinal propulsion changes in SAP rat model treated by DCQD were investigated.

## 2. Materials and Methods

### 2.1. Preparation of DCQD Extract

Four herbs (voucher specimen, number 20110901-4) of DCQD were bought from Chengdu Green Herbal Pharmaceutical Co. Ltd. (Chengdu, China). The crude drugs were identified and the prescription for this study was an aliquot from the same batch. DCQD was boiled twice in distilled water (1 : 12, w/v) for 30 min. The blended supernatants were then lyophilized (yield = 23% w/w, dried extract/crude herb). The dried extract was dissolved in distilled water before use.

### 2.2. Quantitative Control of DCQD Extract

The dried extract was dissolved to 6 g/mL of water solution before experiment. Herbal ratio, marker compounds, and contents were measured by high-pressure liquid chromatography (HPLC). The mobile phase was a mixture of solvent (A) 100% methanol and solvent (B) 0.2% aqueous acetic acid (pH 3.12, 1 : 500, v/v) (A-B: 20 min 36 : 64; 19 min 65 : 35; 21 min 70 : 30). The flow rate was 1.0 mL/min and a ten-minute interval was given between sample injections. The column effluents were simultaneously monitored at 254 nm (for aloe emodin, rhein, chrysophanol, and emodin) and 280 nm (for honokiol, magnolol, naringin, and hesperidin). The reference compounds including rhein, emodin, magnolol, honokiol, aloe-emodin, naringin, hesperidin, and chrysophanol were purchased from the National Institute for the Control of Pharmaceutical and Biological Products (Beijing, China). The over-all intra- and interday variations were less than 5% for all analyses. Their recoveries were >90%. The working solution of DCQD was diluted with saline and used in clinical trial and animal experiments.

### 2.3. Human Studies

After the approval of the ethics committee of Human Research of West China Hospital, 120 patients with SAP were recruited in this study. Inclusion criteria included the following: Acute Physiology and Chronic Health Evaluation (APACHE II) score ≥8, patients enrolled within 72 h of disease onset at our department. Ranson score and APACHE II score were estimated on admission as described [15, 16]. Individuals who had significant medical histories of cardiac, hepatic, renal, pulmonary, digestive, hematological, neurological, endocrine, or psychiatric illness were excluded. Supportive therapy includes fluid management, analgesia, nutritional support, and drug therapy. So far, no positive control drug was recommended in the treatment of SAP to date [17]; patients were divided into the DCQD group and control group quasi-randomly. As we described previously, patients who were admitted on odd days made up the DCQD group (*n* = 70, 35 males) and patients who were admitted on even days comprised the control group (*n* = 50, 20 males). The study was placebo-controlled and the patients have been blinded. Members of the DCQD group received an enema (200 mL) of DCQD solution twice a day for 7 d; the solution was prepared by dissolving freeze-dried powder (0.8 g of crude drugs per kg of body weight). The same regimen was followed in controls, except that the DCQD solution was replaced with 200 mL of saline.

Repeated clinical, radiological, and biochemical assessment was performed on days 3, 7, and 14. At the above time points, organ failure was assessed for six organ systems (respiratory, hepatic, gastrointestinal, cardiovascular, neurological, and renal) by collecting the raw data according to the “a clinically based diagnosis and classification system for acute pancreatitis” [18]. Organ dysfunctions were defined as follows: liver failure, serum bilirubin >34 *μ*mol/L (2 mg/dL), alanine transarninase of more than 2 times the normal value; renal failure, serum creatinine (Cr) >177 *μ*mol/L (2.0 mg/dL), or urinary volume <480 mL/24 h; pulmonary insufficiency, PaO_2_ below 60 mm Hg, and dyspnea with respiration >35/min; systolic blood pressure below 90 mm Hg despite adequate fluid resuscitation, heart rate <54/min or >130/min, and MPA <49 mmHg due to cardiocirculatory insufficiency; brain injury, consciousness, delirium, and coma; gastrointestinal insufficiency, paralysis of intestine, hematemesis or dark stools, erosion, and ulcer; coagulation system injury, disseminated intravascular coagulation or prothrombin time >16 s, activated partial thromboplastin time >45 s, platelets <80 × 10^9^/L, and fibrin <1.5–2.0 g/L.

The primary endpoint was mortality during 60-day follow-up, organ failure, infection rate, and duration of organ failure after randomization. Organ failure duration was defined as the time from onset of any organ failure after randomization until recovery of organ function. Intestinal paralysis release time was defined as the time from treatment until evacuating or defecation for the first time. In this study, infectious events in the course of SAP, including extra-pancreatic or pancreatic infections, were recorded. The incidence of infection was determined, and the relationships between infection with severity indexes, blood biochemical parameters on admission, organ dysfunction during the clinical course, mechanical ventilation, and prognosis were analyzed.

### 2.4. Animal Studies

Animal experiments were approved by the Animal Ethics Committee of Sichuan University and carried out in accordance with their guidelines. Twenty-four male rats weighing 250–300 g were divided into three groups randomly (8 rats in each group) and fasted 12 h before experiments. They were anesthetized with pentobarbitone sodium at a dose of 50 mg/kg (i.p.). A laparotomy was performed. The biliopancreatic duct was cannulated transduodenally with a polyethylene tube according to the method described by Hietaranta et al. [19]. We injected 5% sodium taurocholate solution (taurocholic acid and sodium salt; Sigma Chemical Co., St. Louis, MO, USA) at 0.1 mL/100 g with a constant flow speed. The hepatic portion of the biliopancreatic duct was clipped before injecting the solution. Sham-operated rats were subjected to anesthesia, laparotomy, and duodenal manipulation, but not to biliopancreatic duct cannulation. DCQD or vehicle was administered by gavage 1 h before the model induction. Rats were randomized into three groups: (1) SAP control group, which was given vehicle (0.9% NaCl, 1 mL/kg); (2) SAP + DCQD group, which received DCQD extract (15 g/kg); (3) sham-operated control group. The rat dose of DCQD (15 g/kg) in the present study was converted according to our previous study of human (DCQD, 0.8 g/kg). At 7.5 hours after SAP was induced, rats were gavaged by 2% dextran-2000 (0.4 mL). All rats were executed by the jugular mutilation 30 min after the administration of glucan, and the entire small intestine was removed. Plasma was harvested from the collected blood of abdominal aorta and kept at −20°C for plasma amylase, IL-1*β*, TNF-*α*, and IL-10 assays. Jejunum tissue (50–100 mg) was removed by surgery, immediately snap-frozen in liquid nitrogen, and stored at −80°C for VIP assay. Other parts of the intestine were immediately removed and fixed in the 4% paraformaldehyde for 24 h at room temperature and then processed for immunohistochemistry and hematoxylin and eosin stain.

### 2.5. Plasma Amylase Assays

Plasma amylase levels were determined at 37°C by means of an enzymatic assay (Sigma, St. Louis, MO, USA) with a spectrophotometer according to the manufacturer's instructions. All plasma samples were assayed in duplicate, and the results were averaged at the end of each experiment.

### 2.6. Plasma IL-1*β*, TNF-*α*, and IL-10 Assays

To detect the levels of IL-1*β*, TNF-*α*, and IL-10, blood was placed on ice for 15 minutes and centrifuged at 3000 rpm at 4°C for 10 minutes. Serum levels of IL-1*β*, TNF-*α*, and IL-10 were measured by commercially available enzyme-linked immunosorbent assays (ELISA) (Chemicon, Temecula, CA, USA). According to the manufacturer's protocol, absorbance was measured at 450 nm with High Through Universal Microplate Assay. The sample values were then read off the standard curve. The concentrations were expressed as pg/mL.

### 2.7. Morphometric Studies of the Guts

The intestinal tissues were fixed by 4% formaldehyde. Tissue sections (6 *μ*m thick) were prepared by paraffin embedding. After hematoxylin and eosin (H&E) staining, slides were observed with a light microscope. All the sections were graded by experienced investigators who had no knowledge about the conditions of the specimens. Ten visual fields were observed randomly in a slide under 400x magnification. The histological scoring system of intestinal mucosal damage was classified into five grades.

Briefly, mucosal damage was graded from 0 to 5 according to the following criteria: grade 0, normal mucosal villi; grade 1, development of subepithelial Gruenhagen's space at the apex of the villus with capillary congestion; grade 2, extension of the subepithelial space with moderate lifting of the epithelial layer from the lamina propria; grade 3, massive epithelial lifting down the sides of villi, possibly with a few denuded tips; grade 4, denuded villi with the lamina propria and dilated capillaries exposed, possibly with increased cellularity of the lamina propria; and grade 5, digestion and disintegration of the lamina propria, hemorrhage, and ulceration. The final score for each animal was determined from the means of scores of sections obtained from the terminal jejunum.

### 2.8. Intestinal Propulsion Index

The rats were killed 30 min after the administration of glucan, and the entire small intestine was removed. The small intestine was rapidly dissected out and placed on a clean surface. The small intestine was carefully inspected and the distance traversed by the glucan from the pylorus was measured. The pylorus movement in the intestine was expressed as a percentage. The rate of small intestinal propulsion was calculated by dividing the distance of the intestinal canal labelled by glucan migration by the total length of the small intestine.

### 2.9. Detection of Serum VIP and Tissue VIP

The serum VIP and tissue VIP concentrations were determined with an established available enzyme-linked immunosorbent assay (ELISA) kit (Adlitteram diagnostic laboratories), which measures rat VIP by double-antibody sandwich ELISA. According to the instructions of the manufacturer, the OD 450 was read on a 96-well microplate reader (Tecan Systems, Inc.).

## 3. Statistical Analysis

Data analysis was performed with PEMS 3.1 for windows. All data were expressed as mean ± SD. Differences in clinical parameters between the control and DCQD groups at baseline and different time points were assessed using a *t*-test. One-way ANOVA was used to analyze group differences in the animal studies. Differences with a *P* < 0.05 were considered to be statistically significant. The accepted level of statistical significance for entry of each covariate into logistic regression analysis was 0.05 if the variable was considered clinically relevant. Multivariate logistic regression analysis was used to assess the independent prognostic significance of covariates which were significantly associated with infection.

## 4. Results

### 4.1. Analysis of Chemical Components in DCQD

**Figure 1:**
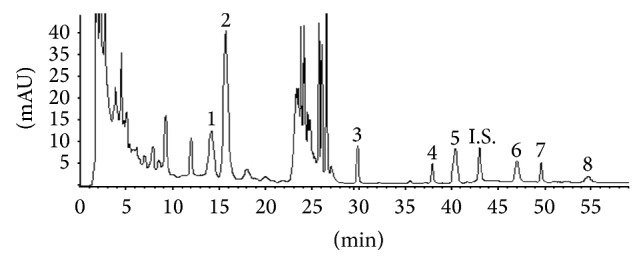
Chromatogram of Da-Cheng-Qi decoction (DCQD) sample (1), hesperidin (2), aloe emodin (3), rhein (4), honokiol (5), magnolol (6), emodin (7), and chrysophanol (8) with I.S.

According to the HPLC method used, the content (mg/g) and retention time (Rt, min) of the eight marker compounds of DCQD were as follows: Rhein 0.88 ± 0.05 (Rt = 37.355), emodin 2.39 ± 0.16 (Rt = 49.060), magnolol 1.06 ± 0.07 (Rt = 47.373), honokiol 1.25 ± 0.08 (Rt = 40.579), Aloe-emodin 1.72 ± 0.08 (Rt = 30.065), naringin 3.89 ± 0.07 (Rt = 14.484), hesperidin 11.27 ± 0.23 (Rt = 16.429), and chrysophanol 0.52 ± 0.03 (Rt = 54.463). The HPLC trace of DCQD is shown in Figure 1.

### 4.2. Human Studies

**Figure 2:**
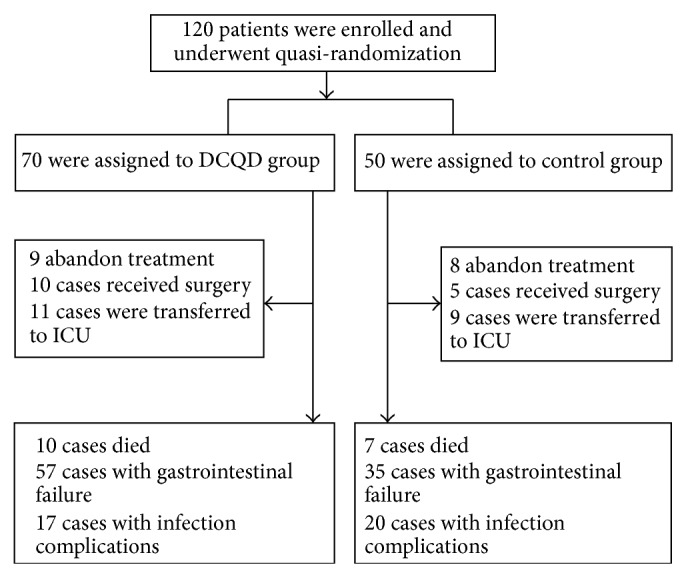
The enrollment, randomization, and followup of this trial.

During the study period of one year, 120 patients were enrolled in the study, but 17 patients were lost to follow-up during the 2 months of trial. The final study was confined to 103 patients. The male : female ratio was 1.18 : 1.00 (65 males : 55 females). Enrollment, randomization, and followup of all subjects in this trial are shown in Figure 2. There were no significant differences in patient characteristics or illness severity among the patients receiving DCQD or placebo at baseline (Table 1). Out of 120 patients, 109 patients had organ dysfunction at admission. The most common organ complication was gastrointestinal injury, which occurred in 75% patients. Clinically detectable signs of lung injury were seen in up to 70% of all the patients. The most severe form of lung injury, adult respiratory distress syndrome (ARDS) occurred in 23 patients. Incidence of other organ failures was cardiocirculatory (43.3%), coagulation system (37.5%), liver (23.3%), and kidney (23%) in turn. Less involved complication is nervous system. In total, 17 patients died in this study. The mortality rate was 14.2%. Five patients in DCQD group had multiple-organ dysfunction syndrome in the early stage. The remaining five patients in the treated group died at one month because of infection and septicemia. Seven patients in the control group died because of cardiopulmonary failure, ARDS, and septic shock. Our clinical outcomes show that DCQD did not affect the overall severity of SAP (based on SOFA scores) or total mortality during two months. The two groups showed no significant differences in early-stage mortality (within 14 days after disease onset) or late-stage mortality (beyond 14 days after onset, up to 2 months). Intensive care and hospital stays did not differ between the two groups.

### 4.3. Duration of Gastrointestinal Dysfunction Was Shortened

**Figure 3:**
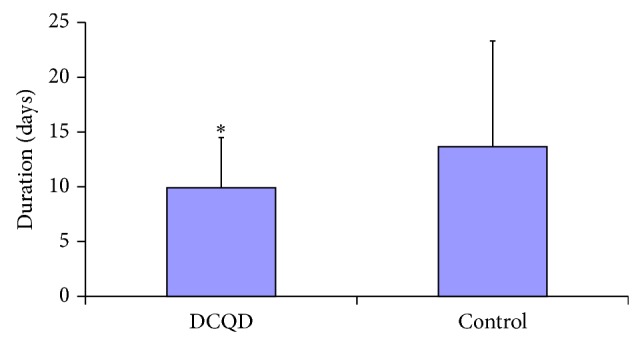
Effect of Da-Cheng-Qi decoction (DCQD) treatment on the mean duration of gastrointestinal failure in severe acute pancreatitis patients. ^*^ *P* < 0.05 versus control group (*n* = 70/DCQD group).

Although DCQD at the dose used in this study did not affect the overall percentage of patients with gastrointestinal failure (81% in DCQD group, compared with 69% in controls *P* > 0.05), it did shorten the average time of the gastrointestinal failure and paralytic intestinal obstruction: the mean duration of gastrointestinal failure in DCQD patients was 9.91 ± 4.59, compared with a mean duration of 13.67 ± 9.65 in controls (*P* < 0.05) (Figure 3).

### 4.4. Infectious Complications Were Decreased

**Figure 4:**
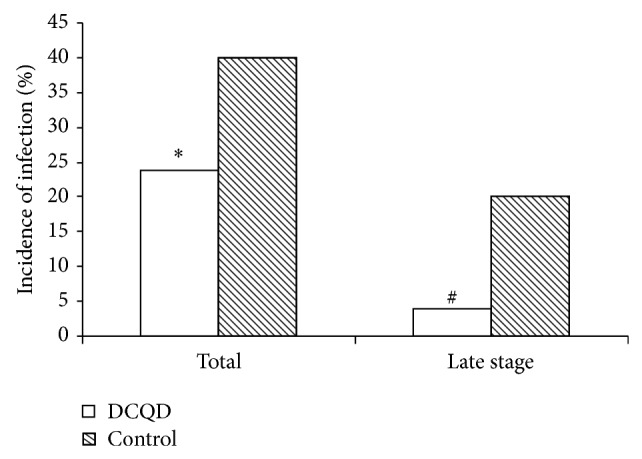
Effect of Da-Cheng-Qi decoction (DCQD) treatment on the total infection incidence or late-stage infection incidence in severe acute pancreatitis patients. ^*^ *P* < 0.05 versus control group (*n* = 70/DCQD group).

The evolution of acute pancreatitis was marked by the occurrence of at least one infectious episode in 35 patients (29.0%). Sixteen patients had infection complications in DCQD group (23.8%), while 20 patients became infected in the control group (40%). It is noticeable that the incidence of the infection was decreased in the treated group (*P* < 0.05). Infectious episode of late stage (beyond 14 days after onset, up to 2 months) is also decreased in DCQD group (4%) compared with the control group (20%) (*P* < 0.05) (Figure 4). The most common documented sites of infections were peritoneal fluid (27.4%), respiratory tract (27.2%), urinary tract (16.1%), intestinal tract (16.1%), and bacteremia (13.0%). Classical risk factors of infection were obtained from a multivariate logistic regression analysis: age, Ranson, Balthazar and APACHE II scores, hospitalization in ICU, local complications, and organ failure (Table 2). The multivariate regression analysis determined the intestinal paralysis release time, hospitalization in ICU, and mechanical ventilation time as infection-related risk factors (*P* < 0.01).

### 4.5. Animal Studies

#### 4.5.1. Plasma Amylase in Rats with SAP

The necrosis of the pancreas leads to elevated levels of plasma amylase in SAP. The level of serum amylase was significantly higher in the SAP model than that in the sham operation group. In rats treated with DCQD, amylase in serum was lower than that that in the control group (*P* < 0.05) (Table 3).

#### 4.5.2. Plasma IL-1*β*, TNF-*α*, and IL-10 Level

The levels of serum IL-1*β*, TNF-*α*, and IL-10 were significantly higher in the SAP group than that in the sham group at the 12 h time points (*P* < 0.05). Compared with the AP group, the levels of IL-1*β* and TNF-*α* decreased in the DCQD treated group (*P* < 0.05) while IL-10 was increased (*P* < 0.01) (Table 4).

### 4.6. Morphological Changes and Pathological Lesion Score in Intestinal Mucosa

**Figure 5:**
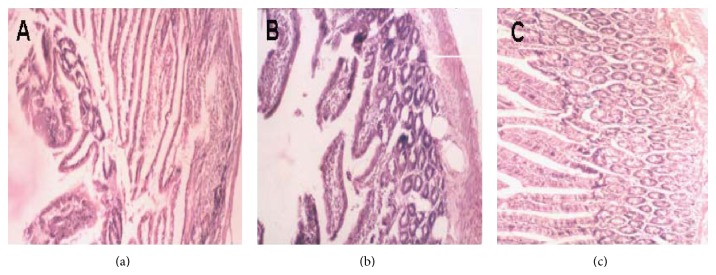
The histopathology changes of small intestine ascending colon by a hematoxylin and eosin (H&E) dyeing: the pathologic changes of intestinal cell swelling, inflammatory cell infiltration, congestion and lamellar hemorrhage even the incomplete structure were observed in SAP. (a) Sham group; (b) SAP group; (c) DCQD group (H&E dyeing ×400).

Pathologically the intestine showed edema, villose exfoliation, degeneration of mucosal cells, mucosal cell necrosis, bleeding, and leukocytic infiltration 12 hours after the SAP model was established. However, the changes in the DCQD group were clearly alleviated compared with those in the AP group (Figure 5). A quantitative score standard for severity was based on pathological changes of the intestinal mucosa in the three groups. As shown in Table 5, the pathological severity scores of the AP and DCQD groups were higher than those in the sham group at 12 hour (*P* < 0.05), while they were lower in the DCQD group than those in the AP group (*P* < 0.05).

### 4.7. Effects on Small Intestinal Propulsion Rate

Although small intestinal propulsion was markedly delayed in SAP model, delayed small intestinal propulsion was improved after the treatment with DCQD (15 g/kg). In sham-operated animals, 30 min after glucan administration, the glucan meal transversed 32.09% of the total length of the small intestine (Table 6). SAP inhibited the small intestinal transit rate compared to that of sham-operated group (*P* < 0.05). When DCQD was administrated in advance, the intestinal propulsive rate increased to 24.66%, which is higher than that of SAP group (*P* < 0.05).

### 4.8. Comparison of Serum VIP and Comparison of Intestinal Tissue VIP in Groups

It was shown that the serum VIP in SAP group was significantly higher than that of sham group (*P* = 0.000), while the serum VIP in DCQD group was lower than that of SAP group after 12 h treatment (*P* = 0.002). The level of tissue VIP in intestine of SAP group was higher than that of sham group (*P* = 0.003), while the level of VIP in DCQD group was lower than that of SAP group (*P* = 0.03) (Table 7).

## 5. Discussion

In our clinical study, we found that DCQD did not affect the overall incidence of patients with gastrointestinal failure, but it did shorten the average time that patients suffered from paralytic intestinal obstruction. The time from DCQD treatment until evacuating or defecation for the first time was shortened. The animal study also demonstrated that DCQD could improve intestinal motility in the SAP rat models with regulating the inflammatory response and alleviating the pathological injury of intestine. The animal experimental findings were consistent with the clinical results, suggesting that DCQD treatment could improve the SAP related intestinal injury significantly.

To determine the mechanism by which the intestinal motility was promoted, further studies were performed in rats' model. We found that rats treated with DCQD showed significantly lower levels of proinflammatory cytokine IL-1*β* and TNF-*α* than those in the SAP group. Previous studies showed that the activation of inflammatory cells is often accompanied by the decrease in electric activity of smooth muscle, which leads to paralysis of intestine. Mediators of inflammation such as tumor necrosis factor alpha (TNF-*α*), interleukin-6 (IL-6), and endothelin-1 (ET-1) are closely related to paralysis of intestine [20, 21]. These results suggested that the effect of DCQD might be restricted to stimuli of cytokine on intestine.

In addition, gastrointestinal motility is mainly affected by neural and endocrine regulations, which are closely related to each other. A number of gastrointestinal hormones played important roles in the regulation of gastrointestinal motility [8, 22]. Our experiment confirms that VIP was increased, which will suppress gastrointestinal motility in the course of SAP. Compared with the SAP group, the levels of VIP in intestinal tissue or serum in DCQD group were obviously lowered, which improve gastrointestinal motility significantly. Another noteworthy phenomenon of beneficial effect of DCQD in human study was that it shortened the average time of the paralytic intestinal obstruction and decreased the infection incidence.

Published data hold that the delayed gastrointestinal motility with systemic and local immunosuppression from the early phase of SAP could cause small intestinal bacterial overgrowth [23]. Bacterial translocation from the digestive tract has been a major source of pancreatic and peripancreatic infection [24]. The important role of intestine motility related infection in SAP was also confirmed in our study: logistic regression analysis showed that intestinal motility (intestinal paralysis release time) was closely related to infection as well as hospitalization in ICU and mechanical ventilation time. This suggested that the intestinal paralysis release time may be a new and useful parameter for prediction of the infectious complication of SAP.

In our study, we found that DCQD did not reduce the mortality but significantly lowered the total infectious complications and the occurrence of late-stage infection from 14 days to 2 months. It is because that the mortality may be due to the other severe complications leading to the death in the early stage of SAP, including ARDS.

On the other hand, we noticed that DCQD did not improve the Ranson's and APACHE II scores, which are the “traditional” multifactorial scoring systems in predicting the severity, pancreatic necrosis, and mortality of AP.

Possible explanations as to why Ranson's and APACHE II scores had not improved may be that the scoring system did not take into consideration the infection and the gastrointestinal injury of the patients. In addition, Ranson's system focused on the data of patients within 48 hours on admission which might be poorly predictive in the late stage of infection occurrence of SAP. These may be some of the reasons for the negative result between these two groups after the treatment of DCQD at the late stage. Limitations of this study were that it is just explanatory study in a single center. It should be designed randomly with blind method with enough time to follow-up. In conclusion, traditional Chinese herbal medicine DCQD appears to increase the intestinal propulsion index, inhibit the production of inflammatory mediators, and alleviate the pathological damage of the intestinal injury.

## Figures and Tables

**Table 1:** The demographic and clinical characteristics of patients at baseline.

	Control	DCQD
Case numbers	50	70
Gender male/female	20/30	35/35
Age (year)	50.94 ± 12.73	49.26 ± 14.37
Ranson	4.38 ± 2.18	4.83 ± 2.11
APACHE II	11.59 ± 6.99	11.33 ± 6.47

Data are the real case number or the mean ± SD. Ranson score and APACHE II score were estimated on admission (*t* < 48 h).

**Table 2:** Multivariate logistic regression analysis of independent risk factors linked to infection.

Risk factors	Odds ratio (CI 95%)	*P*
Intestinal paralysis release time	0.953 (0.914–0.993)	0.023
Hospitalization in ICU	18.525 (6.03–56.914)	0.000
Mechanical ventilation time	0.742 (0.61–0.902)	0.003

**Table 3:** Effect of DCQD on plasma amylase in SAP rats (*n* = 8).

	Sham	Control	DCQD
AMY (IU·L^−1^)	992 ± 221	2077 ± 585^*^	1458 ± 450^#^

^*^ *P* < 0.05 versus sham operation group. ^#^ *P* < 0.05 versus control group.

**Table 4:** Serum levels of IL-1*β*, TNF-*α*, and IL-10 obtained through ELISA in different groups with and without being treated by Da-Cheng-Qi decoction (DCQD) (*n* = 8) (pg/mL).

	IL-1*β*	TNF-*α*	IL-10
Sham	0.26 ± 0.13	1.57 ± 0.13	39.2 ± 8.98
Control	0.61 ± 0.17^*^	2.96 ± 0.29^*^	48.4 ± 10.51^*^
DCQD	0.30 ± 0.04^#^	2.40 ± 0.20^#^	67.8 ± 20.01^#^

^*^ *P* < 0.05 versus sham operation group. ^#^ *P* < 0.05 versus control group.

**Table 5:** Pathological score in SAP rats' intestinal tissue with and without being treated by Da-Cheng-Qi decoction (DCQD) (*n* =8) (mean ± SD).

	Sham	Control	DCQD
Pathological score	0.15 ± 0.03	4.3 ± 1.5^*^	2.2 ± 0.8^#^

^*^ *P* < 0.05 versus sham operation group. ^#^ *P* < 0.05 versus control group.

**Table 6:** Comparison of intestinal propulsion rate (%) (*n* = 8).

	Sham	Control	DCQD
IPR	32.09 ± 6.65	18.30 ± 3.82^*^	24.66 ± 4.54^#^

^*^ *P* < 0.05 versus sham operation group. ^#^ *P* < 0.05 versus control group.

**Table 7:** Comparison of serum VIP and comparison of intestinal tissue VIP in groups (ng/mL) (*n* = 8).

	Sham	Control	DCQD
Serum VIP	10.19 ± 2.25	18.75 ± 3.84^a^	13.8 ± 2.7^b^
Tissue VIP	2.1 ± 0.65	3.03 ± 0.74^c^	2.36 ± 0.32^d^

^a^VS sham serum group; ^b^VS control serum group; ^c^VS sham tissue group; ^d^VS control tissue group.
